# Characterization of the complete chloroplast genome of *Arabis stellari* and comparisons with related species

**DOI:** 10.1371/journal.pone.0183197

**Published:** 2017-08-15

**Authors:** Gurusamy Raman, Veronica Park, Myounghai Kwak, Byoungyoon Lee, SeonJoo Park

**Affiliations:** 1 Department of Life Sciences, Yeungnam University, Gyeongsan, Gyeongsan-buk, Republic of Korea; 2 Mcneil high school, Austin, Texas, United States of America; 3 Plant Resources Division, National Institute of Biological Resources of Korea, Incheon, Republic of Korea; National Renewable Energy Laboratory, UNITED STATES

## Abstract

*Arabis stellari* var. *japonica* is an ornamental plant of the Brassicaceae family, and is widely distributed in South Korea. However, no information is available about its molecular biology and no genomic study has been performed on *A*. *stellari*. In this paper, the authors report the complete chloroplast genome sequence of *A*. *stellari*. The plastome of *A*. *stellari* was 153,683 bp in length with 36.4% GC and included a pair of inverted repeats (IRs) of 26,423 bp that separated a large single-copy (LSC) region of 82,807 bp and a small single-copy (SSC) region of 18,030 bp. It was also found to contain 113 unique genes, of which 79 were protein-coding genes, 30 were transfer RNAs, and four were ribosomal RNAs. The gene content and organization of the *A*. *stellari* chloroplast genome were similar to those of other Brassicaceae genomes except for the absence of the *rps16* protein-coding gene. A total of 991 SSRs were identified in the genome. The chloroplast genome of *A*. *stellari* was compared with closely related species of the Brassicaceae family. Comparative analysis showed a minor divergence occurred in the protein-coding *matK*, *ycf1*, *ccsA*, *accD* and *rpl22* genes and that the K_A_/K_S_ nucleotide substitution ratio of the *ndhA* genes of *A*. *stellari* and *A*. *hirsuta* was 1.35135. The genes *infA* and *rps16* were absent in the *Arabis* genus and phylogenetic evolutionary studies revealed that these genes evolved independently. However, phylogenetic analysis showed that the positions of Brassicaceae species are highly conserved. The present study provides *A*. *stellari* genomic information that may be found useful in conservation and molecular phylogenetic studies on Brassicaceae.

## Introduction

Chloroplasts are the most noticeable feature in green plant cells and are specific to plants. The chloroplast is a semi-autonomous organelle that was derived from a cyanobacterial endosymbiont around one billion years ago [1, 2]. Plastids are involved in several critical biochemical processes other than photosynthesis, such as, starch biosynthesis, nitrogen metabolism, sulfate reduction, fatty acid synthesis, and DNA and RNA synthesis [3]. The high copy number of plastomes in plant cells is inherited maternally in most plant cells, and the chloroplast genome varies in size from 75 to 250 kb and is highly conserved in terms of gene contents and genome structure in vascular plants [4, 5]. Chloroplasts are normally separated by two large inverted repeat regions separated by a large single-copy region (LSC) and small single-copy region (SSC) that vary in length. Currently, more than 1100 genomes are available in the chloroplast genome database. Comparative studies on these genomes have shown some infrequent structural changes, such as, gene or intron loss, large inverted repeat (IR) expression, inversions and rearrangements in many land plants [6]. For example, intron loss was observed in the *clpP* gene of *Sileneae* [7], *infA* gene loss in Brassicales, Cucurbitales, Fabales, Fagales, Malphighlales, Malvales, Myrtales, Rosales, Sapindales, Solanales, *Dianthus*, and *Lychinis* [8–12], *rpl22* gene loss in Fagaceae and Passifloraceaae [13], *rpl23* loss in *Dianthus*, *Lychnis* and *Spinacia* [12, 14], *rpl32* gene loss in *Populus* [15], *ycf2* gene loss in rice and maize [16, 17], and *ycf4* gene loss in all legume plants of angiosperms [18, 19]. Such studies provide information for plant phylogenetic tree reconstruction [20], DNA barcoding [21], and for population [22], transplastomic, and evolutionary studies [23].

The herbaceous Brassicaceae plants are distributed worldwide. They Brassicaceae family is composed of more than 3700 species, and includes vegetable and vegetable oil crops, ornamentals, and model species [6]. The ornamental plant, *A*. *stellari* var. *japonica* also belong to this family and is widely distributed in Russia, Taiwan, Japan, and South Korea. It grows up to a height of 30 centimeters, is sparsely to densely pilose, has erect or ascending stems, is basal and cauline, and it a popular garden plant. To the best of our knowledge, no previous molecular or genomic study has been carried out in this ornamental plant and its plastome sequence has not been reported. In the present study, we sought to determine the complete chloroplast genome sequence of *A*. *stellari*, to describe the structure of the plastome genome, and to compare its plastome genome with those of closely related Brassicaceae species. Accordingly, we sought to expand understanding of the diversity of *Arabis* chloroplast genomes and provide basic data for phylogenetic studies on Brassicaceae.

## Materials and methods

### DNA extraction and sequencing

The *A*. *stellari* plant sample was collected on Dokdo island (South Korea). DNA was extracted using a modified CTAB method [24]. Whole-genome sequencing was performed using Illumina NextSeq 500 (LabGenomics, South Korea) technology and a paired-end library of 2x101 bp and insert size of ~200 bp. About 152,770,066 raw reads were trimmed and filtered using Genious v10.1 (Biomatters, New Zealand). Filtered reads were assembled using *A*. *alpina* (NC_023367) as a reference genome. Consensus sequences were extracted and specific primers were designed based on gaps between sequences and these gaps were filled by polymerase chain reaction (PCR) amplification. PCR products were purified and sequenced using the conventional Sanger sequencing method. The chloroplast genome sequencing data and gene annotation were submitted to GenBank and assigned the accession number KY126841.

### Chloroplast genome annotation and sequence statistics

The online program Dual Organeller GenoMe Annotator (DOGMA) was used to annotate the *A*. *stellari* cp genome [25]. The initial annotation results were checked manually and putative starts, stops, and intron positions were adjusted by comparing them with closely related homologous genes of *A*. *alpina*, *A*. *hirsuta*, and *Arabidopsis thaliana*. Transfer RNA genes were verified using tRNAscan-SE version1.21 and default settings [26]. The OGDRAW program was used to draw a circular map of the *A*. *stellari* cp genome [27].

### Comparative genome analysis

The mVISTA program in Shuffle-LAGAN mode was used to compare the *A*. *stellari* cp genome with four other cp genomes using *A*. *stellari* annotation as a reference [28]. The boundaries between IR and SC regions of these species were also compared and analyzed.

### Analysis of repeat sequences and single sequence repeats (SSR)

REPuter software was used to identify the presence of repeat sequences, including forward, reverse, palindromic, and complementary repeats in the cp genome of *A*. *stellari* [29]. The following conditions were used to identify repeats in REPuter: (1) Hamming distance 3, (2) minimum sequence identity of 90%, (3) and a repeat size of more than 30 bp. Phobos software v1.0.6 was used to detect SSRs of cp genome; parameters for match, mismatch, gap, and N positions were set at 1, -5, -5 and 0, respectively [30].

### Characterization of substitution rates

To analyze synonymous (K_S_) and nonsynonymous (K_A_) substitution rates, the *A*. *stellari* cp genome was compared with the cp genome sequences of *A*. *alpina* and *A*. *hirsuta*. Similar individual functional protein-coding gene exons were extracted and aligned separately using Geneious v10.1.3. Aligned sequences were translated into protein sequences and K_S_ and K_A_ rates were estimated using DnaSP software v5.10.01 [31].

### PCR amplification of the *rps16* gene

The genomic DNA of *A*. *stellari* was used as a template to detect the *rps16* gene and gene specific primers were designed using Primer3 v0.4.0 [32]. The *rps16* gene was amplified using the primers (*rps16*F: 5'–ACCAAGCTATATACGAGTCTTTCA–3' and *rps16*R: 5'-ACGATATACTGACTGAACTATGACT–3'), and the PCR product was purified using the Solg^™^ Gel & PCR purification System Kit (Solgent Co., Daejeon, South Korea). Purified PCR products were sequenced with an ABI 3730XL DNA analyzer (Applied Biosystems, Foster City, USA) at Solgent. The nucleotide sequence of *rps16* was aligned using MAFFT v7 [33] in Geneious v10.1.3 (Biomatters, New Zealand).

### Phylogenetic analysis

A phylogenetic tree was constructed using 76 protein-coding genes of 20 cp genomes of angiosperms using the *Vitis* set as the outgroup. The 19 completed cp genome sequences were downloaded from the NCBI Organelle Genome Resource database S1 Table. *rps16*, *ycf15*, and 76 protein-coding gene sequences were aligned separately using MAFFT v7 [33] through Geneious v10.1.3. The aligned individual gene sequences and protein-coding gene sequences were saved in PHYLIP format using Clustal X v2.1 [34] and phylogenetic analysis was performed based on maximum likelihood (ML) analysis using the general time-reversible model and the gamma model site heterogeneity (GTRGAMMA) nucleotide substitution model using default parameters in RAxML v. 7.2.6 [35]. The bootstrap probability of each branch was calculated using 1000 replications.

## Results and discussion

### Genome organization and features of the *A*. *stellari* cp genome

**Fig 1:**
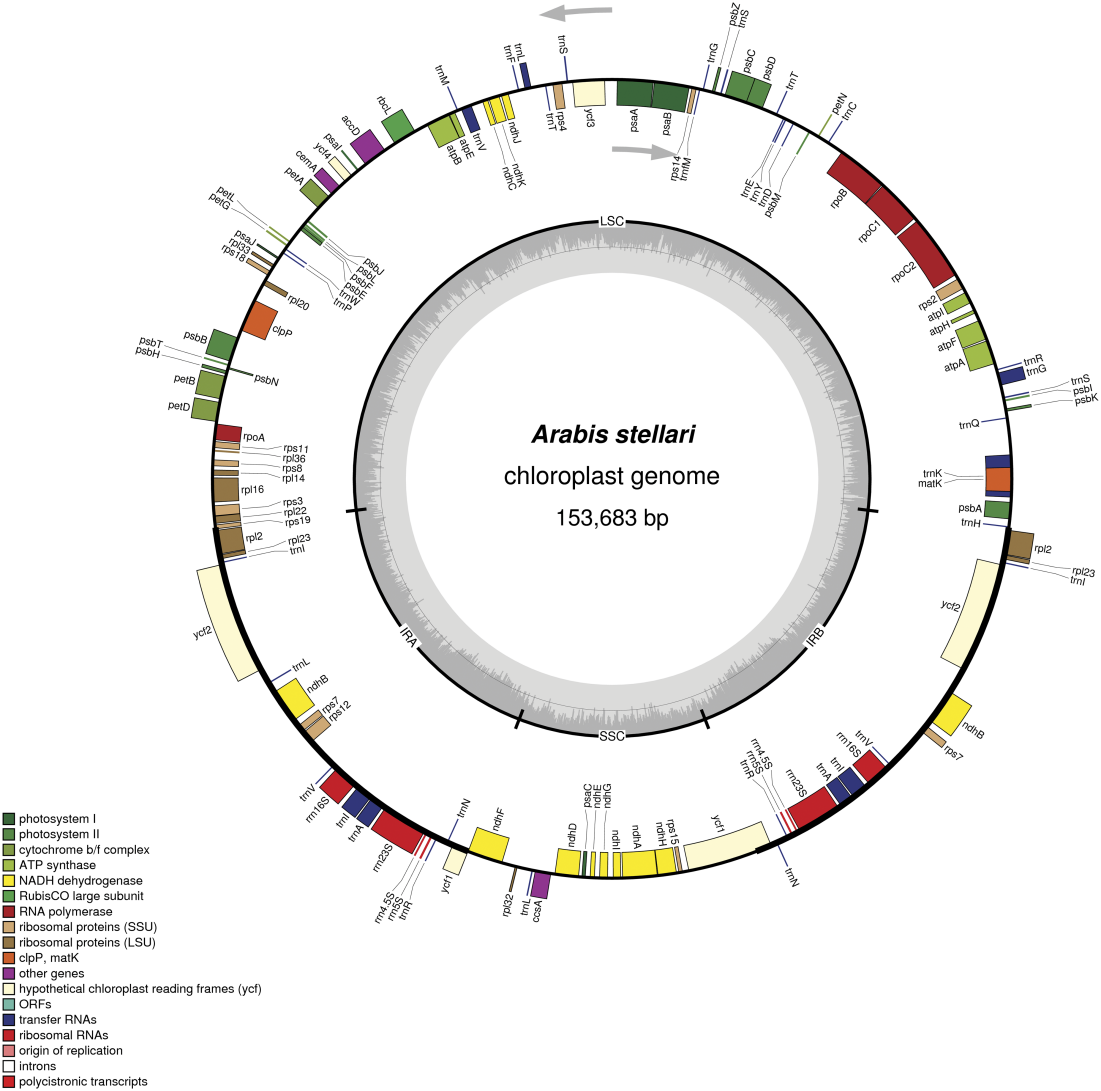
Gene map of *Arabis stellari* var. *japonica*. Genes lying outside of the outer layer circle are transcribed in a counterclockwise direction, whereas genes inside this circle are transcribed in a clockwise direction. The colored bars indicate known protein-coding genes, tRNA genes, and rRNA genes. The dashed darker gray area in the inner circle denotes GC content, while the lighter gray area indicates genome AT content. LSC, large-single-copy; SSC, small-single-copy; IR, inverted repeat.

The complete chloroplast genome of *A*. *stellari* was found to have a total length of 153,683 bp, with a pair of inverted repeats (IRs) of 26,423 bp that separated a large single copy (LSC) region of 82,807 bp and a small single copy (SSC) region of 18,030 bp (Fig 1). Total GC content was 36.4%, which is similar to those of *A*. *alpina* [36], *Draba nemorosa*, and *Brassica napus* [37] whereas GC contents are low in the species *A*. *hirsuta* (33.0%) and *Arabidopsis thaliana* (32.1%) [38]. These results suggest that GC contents are unevenly distributed in the genomes of the Brassicaceae family. In *A*. *stellari*, GC content was higher in the IRs region (42.4%) than in the LSC and SSC region (34.1% and 29.2%). The high GC content percentage in IR regions was attributed to the presence of high GC nucleotide percentages in the four rRNA genes *rrn4*.*5*, *rrn5*, *rrn16*, and *rrn23*. Identical results have been reported for other chloroplast genomes [39, 40].

The chloroplast genome of *A*. *stellari* encoded a total of 113 unique genes, of which 18 were duplicated in IR regions. Of the 113 genes, 79 were protein-coding genes, 30 were transfer genes and four were rRNA genes Table 1. Of these, 14 genes encoded one intron (eight protein-coding and six tRNA genes) and three encoded two introns (*clpP*, *ycf3* and *rps12*). The *rps12* gene was found to be a trans-spliced gene with its 5'- end exon located in the LSC region and its intron 3'-end exon duplicated in IR regions.

**Table 1:** List of genes present in the cp genome of *Arabis stellari*.

Category	Group of genes	Name of genes				
RNA genes	Ribosomal RNA genes	*rrn4*.*5*^a^	*rrn5*^a^	*rrn16*^a^	*rrn23*^a^	
	Transfer RNA genes	*trnA*-UGC^a^^,^ ^b^	*trnC*-GCA	*trnD*-GUC	*trnE*-UUC	*trnF*-GAA
		*trnfM*-CAU	*trnG*-GCC^b^	*trnG*-UCC	*trnH*-GUG^a^	*trnI*-CAU^a^
		*trnI*-GAU^a^^,^ ^b^	*trnK*-UUU	*trnL*-CAA^a^	*trnL*-UAA^b^	*trnL*-UAG
		*trnM*-CAU	*trnN*-GUU^a^	*trnP*-UGG	*trnQ*-UUG	*trnR*-ACG^a^
		*trnR*-UCU	*trnS*-GCU	*trnS*-GGA	*trnS*-UGA	*trnT*-GGU
		*trnT*-UGU	*trnV*-GAC^a^	*trnV*-UAC^b^	*trnW*-CCA	*trnY*-GUA
Protein genes	Subunits of photosystem I	*psaA*	*psaB*	*psaC*	*psaI*	*psaJ*
		*ycf3*^c^	*ycf4*			
	Subunits of photosystem II	*psbA*	*psbB*	*psbC*	*psbD*	*psbE*
		*psbF*	*psbH*	*psbI*	*psbJ*	*psbK*
		*psbL*	*psbM*	*psbN*	*psbT*	*psbZ*
	Subunits of cytochrome	*petA*	*petB*^b^	*petD*^b^	*petG*	*petL*
		*petN*				
	Subunits of ATP synthase	*atpA*	*atpB*	*atpE*	*atpF*^b^	*atpH*
		*atpI*				
	Large subunit of Rubisco	*rbcL*				
	Subunits of NADH dehydrogenase	*ndhA*^b^	*ndhB*^a^^,^ ^b^	*ndhC*	*ndhD*	*ndhE*
		*ndhF*	*ndhG*	*ndhH*	*ndhI*	*ndhJ*
		*ndhK*				
	ATP-dependent protease subunit P	*clpP*^c^				
	Chloroplast envelope membrane protein	*cemA*				
Ribosomal proteins	Small subunit of ribosome	*rps2*	*rps3*	*rps4*	*rps7*^a^	*rps8*
		*rps11*	*rps12*^a^^,^ ^c^^,^ ^d^	*rps14*	*rps15*	*rps16*^e^
		*rps18*	*rps19*			
Transcription	Large subunit of ribosome	*rpl2*^a^	*rpl14*	*rpl16*^b^	*rpl20*	*rpl22*
		*rpl23*	*rpl32*	*rpl33*	*rpl36*	
	DNA-dependent RNA polymerase	*rpoA*	*rpoB*	*rpoC1*^b^	*rpoC2*	
Other genes	Maturase	*matK*				
	Subunit of acetyl-CoA	*accD*				
	C-type cytochrome synthesis gene	*ccsA*				
	Component of TIC complex	*ycf1*^a^				
	Hypothetical proteins	*ycf2*^a^, *ycf15*^a^^,^ ^e^				

^a^—Two gene copies in IRs;

^b^—Gene containing a single intron;

^c^—Gene containing two introns;

^d^—Gene divided into two independent transcription units;

^e^—Pseudogene.

In the total *A*. *stellari* cp genome, protein-coding regions accounted for 79,437 bp (51.68%), intron regions for 19,688 bp (12.82%) and tRNA and rRNA regions for 2,785 bp (1.81%) and 9,049 bp (5.89%) respectively. The remaining regions were intergenic spacers (42,724 bp, 27.8%). The pseudogene, *rps16* was identified in the LSC region. Overall, the gene order and gene contents of *A*. *stellari* were identical to those of *A*. *alpina* and *A*. *hirsuta*.

### Comparisons of the *A*. *stellari* cp genome and those of other Brassicaceae species

The cp genome of *A*. *stellari* was compared with four closely related Brassicaceae family cp genomes, namely with those of *A*. *alpina*, *A*. *hirsuta*, *Brassica napus*, and *A*. *thaliana*. The organization of the Brassicaceae cp genome is highly conserved, and neither translocations nor inversions were identified in the analyses. However, two dissimilarities were identified involving the protein-coding genes *rps16* and *ycf15*, and some differences between total genome sizes were detected. The shortest genome was that of *Brassica napus* (152,860 bp) and the longest that of *Pugionium dolabratum* (155,002 bp). These differences were largely due to variabilities in the length of the LSC region. Similar genome size variations in the LSC region were observed in rosid chloroplast genomes [12].

**Fig 2:**
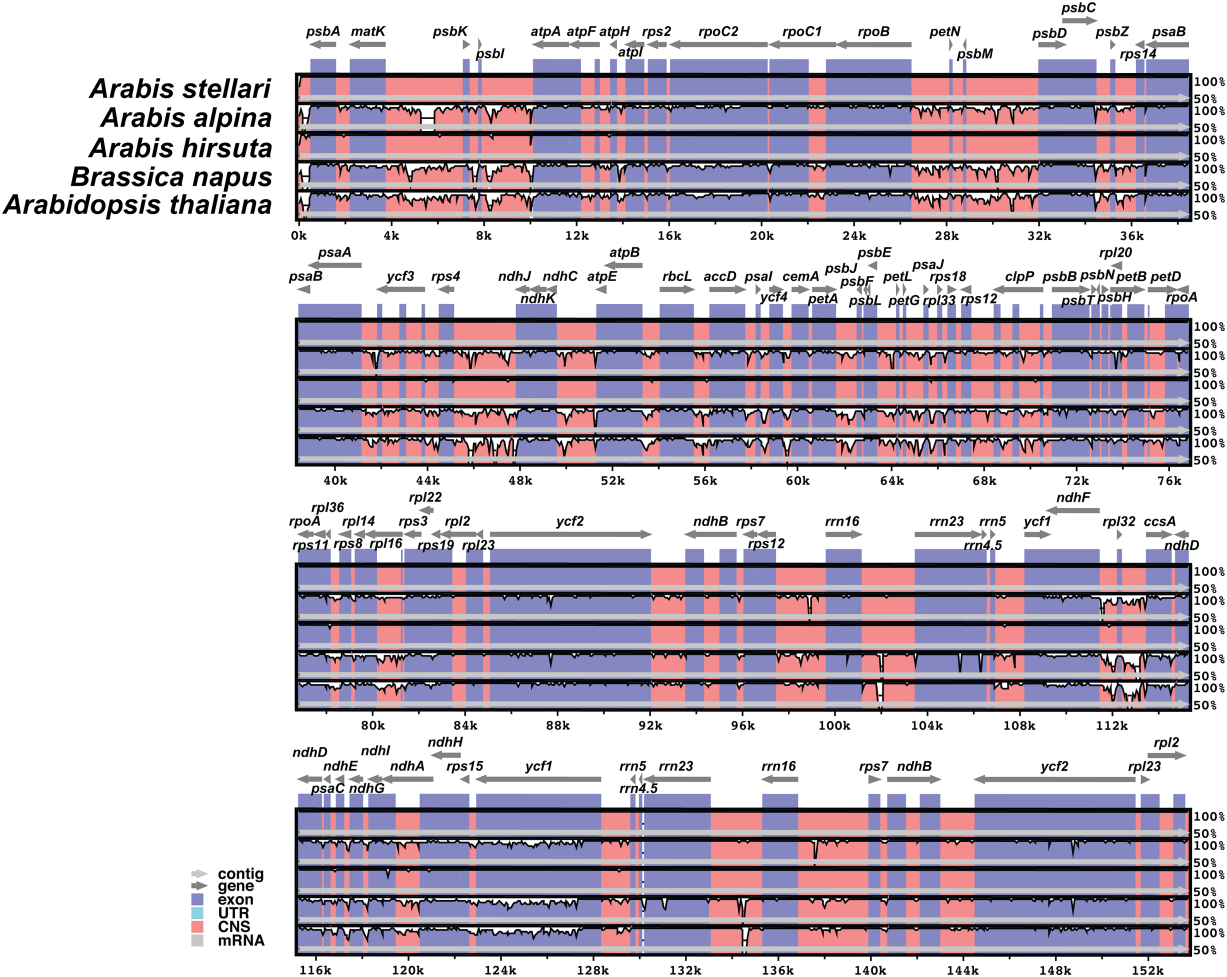
Sequence alignment of six chloroplast genomes in the Brassicaceae family performed using the mVISTA program with *Arabis stellari* var. *japonica* as reference. The top gray arrow shows genes in order (Transcriptional direction) and the position of each gene. A 70% cut-off was used for the plots. The Y-scale represents the percent identity between 50–100%. Red and blue areas indicate intergenic and genic regions, respectively.

**Fig 3:**
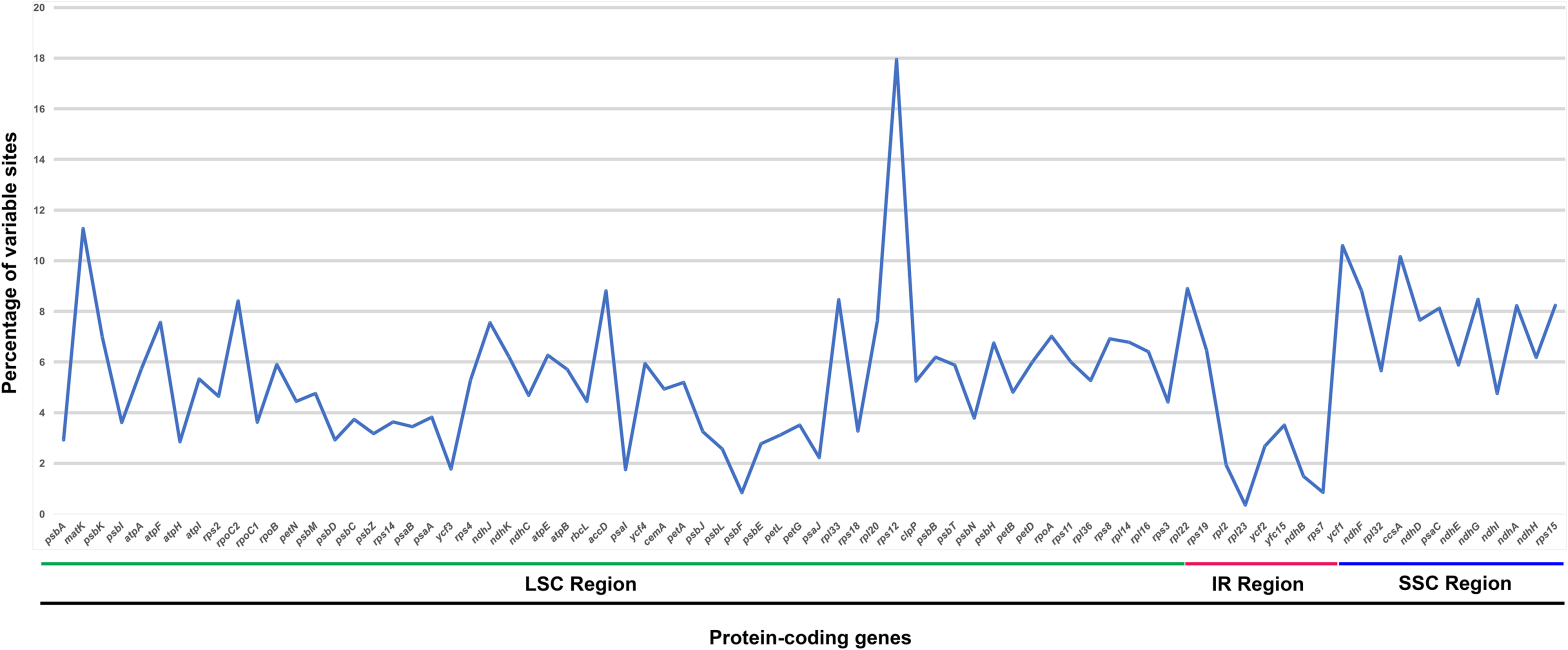
Percentages of variable sites in protein-coding regions across the six Brassicaceae family chloroplast genomes.

The overall sequence variation of five Brassicaceae family cp genomes was plotted using the mVISTA program, and the results obtained revealed that cp genomes within Brassicaceae are highly conserved (Fig 2). However, minor divergences were detected in protein-coding regions. In order to analyze divergent hotspot regions further, all coding regions of *A*. *stellari*, *A*. *alpina*, and *A*. *hirsuta* were extracted and evaluated. The most divergent regions found were in the protein coding genes *matK*, *ycf1*, *ccsA*, *accD*, and *rpl22* (Fig 3), which are present in the large and single copy regions.

**Fig 4:**
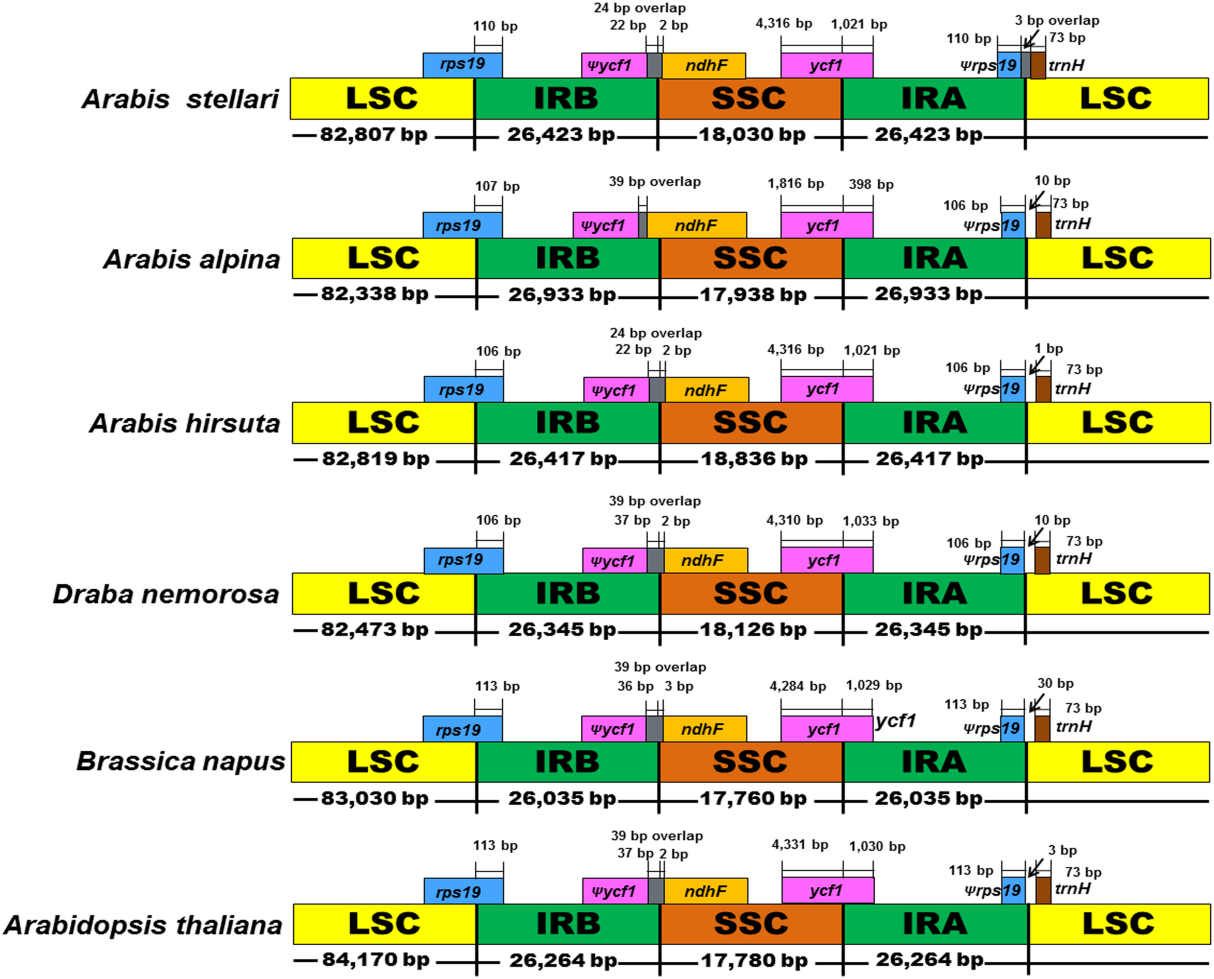
Comparison of the borders of the LSC, SSC, and IR regions of Brassicaceae chloroplast genomes. Indicates a pseudogene. The figure is not drawn to scale.

Due to the size variation exhibited by angiosperm chloroplast genomes, expansion and contraction at IR/SC borders are more common in chloroplast genomes [41]. In the present study, the LSC/IRb/SSC/IRa junctions of the five Brassicaceae family chloroplast genomes were compared (Fig 4). The lengths of the LSC, IR and SSC regions were similar in the cp genomes of *A*. *stellari*, *A*. *alpina*, *A*. *hirsuta* and *D*. *nemorosa* as compared with *B*. *napus* and *A*. *thaliana*; although some variances in IR expansions and contractions were detected. The *rps19* gene was present in the LSC region and expanded in the IR region in all six cp genomes. Also, the pseudogene *ycf1* was completely present in the IR region. Likewise, the *ndhF* genes of *A*. *stellari*, *A*. *hirsuta*, *D*. *nemorosa*, *B*. *napus* and *A*. *thaliana* were completely contained in the SSC region. Whereas the *ndhF* gene of *A*. *alpina* was extended and overlapped with pseudogene *ycf1* in the IRb region. Similarly, the tRNA gene, *trnH*-*GUG* was entirely positioned in IRa region of all chloroplast genomes except that of *A*. *stellari*. Nevertheless, 3 bp of the *trnH* gene in *A*. *stellari* overlapped the IRa region.

### Repeat and SSR analysis

**Fig 5:**
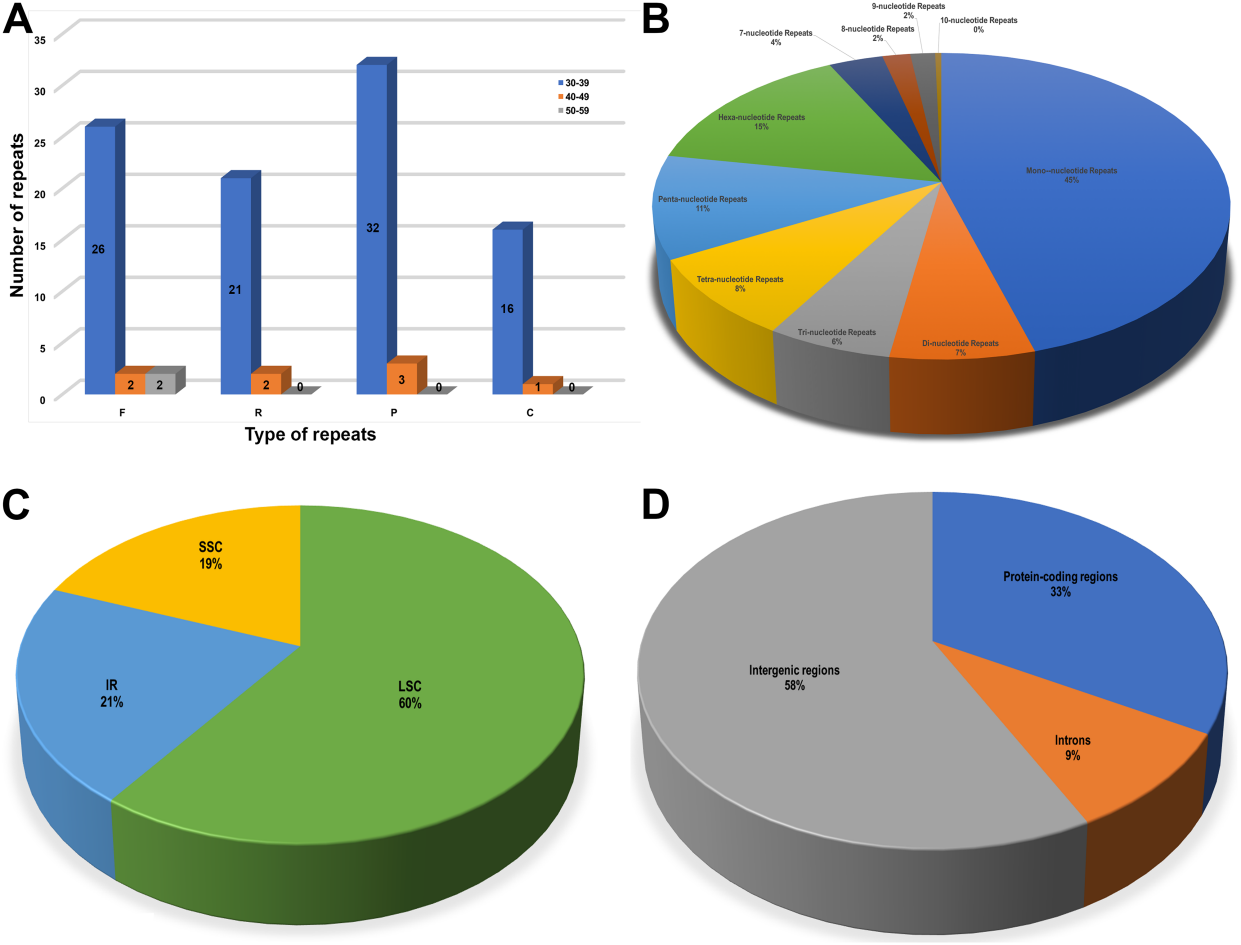
The distribution, types, and presence of simple sequence repeats (SSRs) in the cp genome of *Arabis stellari* var. *japonica*. **(A)** Number of different types of repeats. F—forward repeats; R—Reverse repeats; P—palindromic repeats; C—complement repeats. Presence of SSRs in the LSC, SSC, and IR regions. **(B)** Numbers of different types of SSRs. **(C)** Presence of SSRs in the LSC, SSC, and IR regions. **(D)** Presence of SSRs in protein-coding regions, intergenic spacers, and intron regions.

The REPuter program was used to screen repeat sequences in the *A*. *stellari* chloroplast genome. The results obtained showed the following were present; 30 forward repeats, 23 reverse repeats, 35 palindromic repeats, and 17 complementary repeats (Fig 5A). Of these repeats, 95 (90.5%) were 30–39 bp long, 8 (7.6%) were 40–49 bp long, and 2 (1.9%) were 50–59 bp long. The longest repeat had a length of 56 bp. Simple sequence repeats (SSRs) play significant roles during genome rearrangement and recombination [42]. A total of 991 SSRs were detected in the *A*. *stellari* chloroplast genome (Fig 5B). Of these, 451 (45%) were mono-nucleotide repeats, 69 (7%) di-nucleotide repeats, 60 (6%) tri-nucleotide repeats, 84 (8%) tetra-nucleotide repeats, 108 (11%) penta-nucleotide repeats, 146 (15%) hexa-nucleotide repeats, and 35, 18, 16 and 4 were 7-, 8-, 9- and 10- nucleotide repeats respectively. Of the 991 SSRs, 60% (594), 21% (208), and 19% (189) SSRs were present in the LSC, IR, and SSC regions, respectively (Fig 5C). In addition, we determined number of repeats in protein-coding and intron and intergenic regions (IGS) (Fig 5D), and found 570 (58%), 329 (33%), and 92 (9%) SSRs were located in IGS, protein-coding, and intron regions, respectively. The presence of repeat sequences in the chloroplast genome of *A*. *stellari* may be useful for developing lineage-specific markers for genetic diversity and evolutionary studies.

### Pseudogenization of *rps16* gene

**Fig 6:**
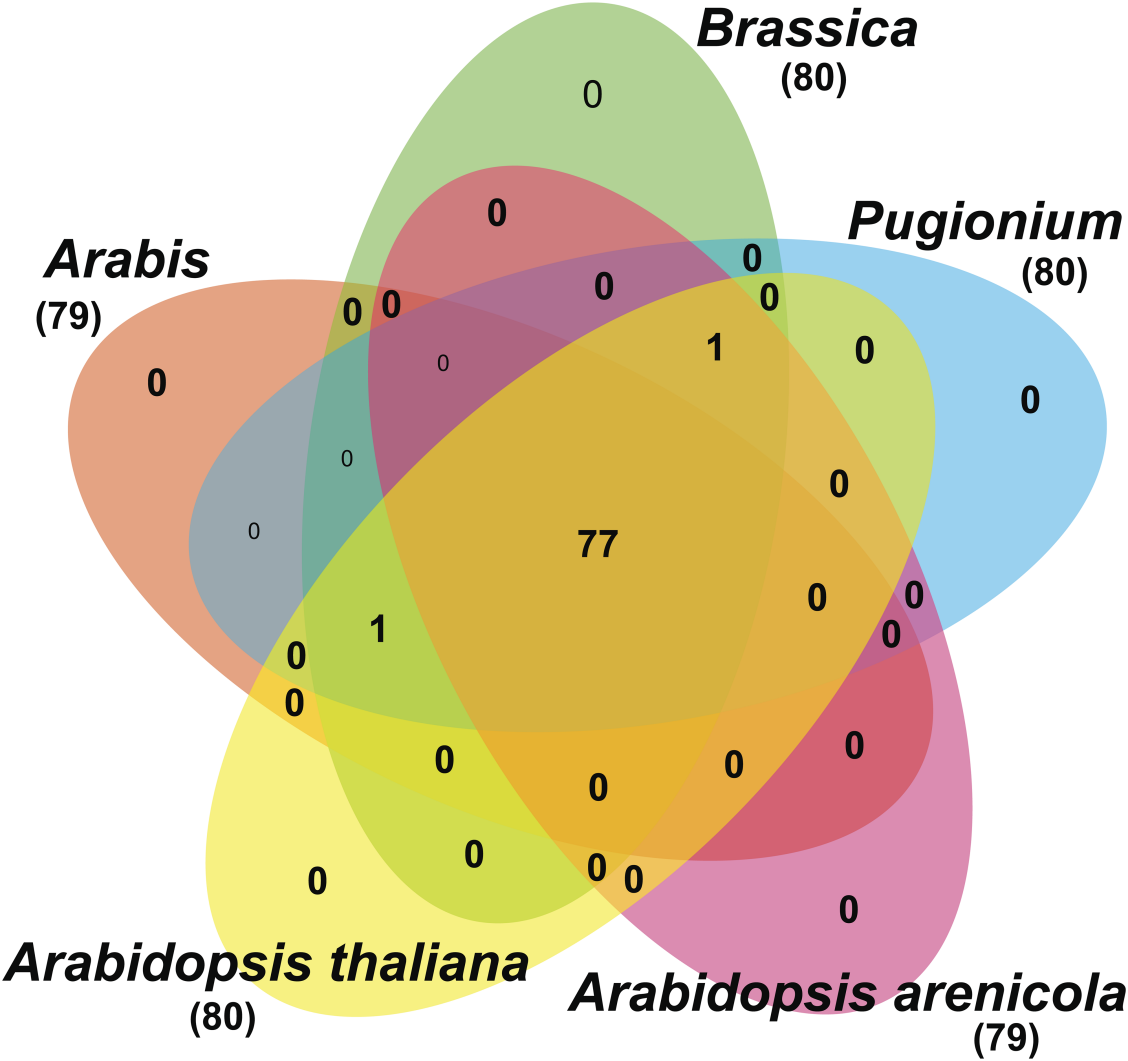
Venn diagram showing the full complement of genes present in sequenced Brassicaceae family chloroplast genomes. tRNAs and rRNAs are not included. Numbers below each species represent the total number of unique protein-coding genes used in the comparison.

In photosynthetic plants, chloroplast gene loss infrequently occurs, but only when nuclear and/or mitochondrial genomes encode another functional copy or acquire one from the plastome through gene transfer [43]. Although the number of genes and their order are generally conserved among angiosperm chloroplast genomes [44]. Besides, rare cases have been observed in the chloroplast genomes of Brassicaceae family [6]. Hence, the cp genome size, %GC content and total number of unique protein-coding genes, tRNA and rRNA genes of 14 Brassicaceae family genomes were compared for analysis of gene duplication, pseudogene or gene deletion in its closely related species of *Arabis* chloroplast genome S2 Table. However, some dissimilarity was identified in protein-coding genes of Brassicaceae. The cp genomes of *Arabis* genus, *D*. *nemorosa*, *Arabidopsis arenicola*, *A*. *arenosa* and *A*. *cebennensis* were found to encode 79 protein-coding genes, whereas *Brassica* genus and *A*. *thaliana* possessed 80 protein-coding genes (Fig 6). This one gene variation was caused by either pseudogenization of *rps16* in the LSC region of the *Arabis* or, pseudogenization of *ycf15* in *A*. *arenicola*, *A*. *arenosa* and *A*. *cebennensis* cp genomes.

The *rps16* gene is critical for cell viability [45] and is involved in the assembly of the 30S subunit [46] in *Escherichia coli*. In order to analyze pseudogenization of the *rps16* gene, we designed a primer and amplified the *rps16* gene of *A*. *stellari* (S1 Fig). The gene sequence of *rps16* confirmed that the *A*. *stellari* chloroplast genome encoded a pseudogene *rps16*. In addition, the *rps16* gene was analyzed and compared with Brassicaceae family chloroplast genomes. Among, 14 Brassicaceae, the *rps16* gene was found to be a pseudogene in *A*. *stellari*, *A*. *hirsuta*, and *D*. *nemorosa* but to be entirely missing in *A*. *alpina* (S2 Fig). The intact nucleotide sequence of *rps16* is ~1,161 bp long which includes two exons (~45-bp—exon I and ~226-bp—exon II) and one intron sequence (~890-bp). In the chloroplast genomes of *A*. *stellari*, *A*. *hirsuta*, and *D*. *nemorosa*, 10-bp deletion within the first exon of *rps16*, leading to a framshift (S2 Fig). Although, deletion of 9-bp found in the second exon of *rps16* of *A*. *stellari*, *A*. *hirsuta*, and *D*. *nemorosa*. Whereas, the *rps16* gene of *A*. *alpina* encoded 21-bp only and it lost the entire second exon and part of the intron sequences. Interestingly, the expression of *rps16* gene analyzed in *A*. *thaliana* cp genome and identified that the cp *rps16* is a pseudogene in this species due to the splicing of the group II intron is defective [10]. Whereas, its closely related species *A*. *arenosa*, *A*. *lyrata* and *Crucihimalaya lasicarpa* were compared and detected that *rps16* is a functional gene in these species. These results suggested that the pseudogenization event must have occurred after the divergence of *Arabidopsis* and its close relatives of Brassicaceae.

**Fig 7:**
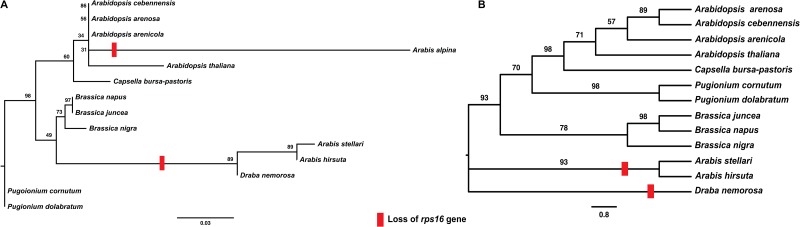
Molecular phylogenetic tree analysis of cp protein-coding gene *rps16* of Brassicaceae family. A. Phylogenetic tree constructed with *Arabis alpina* B. Phylogenetic tree constructed without *A*. *alpina*. Trees were constructed by maximum likelihood (ML) analysis using the RaxML program and the GTRGAMMA nucleotide model. The stability of each tree node was tested by bootstrap analysis with 1000 replicates.

In addition, evolution of the *rps16* gene of *A*. *stellari* accessed by comparing it with 13 other Brassicaceae chloroplast genomes. Phylogenetic analysis showed intron loss of *rps16* in different genus formed one clade and complete gene loss of *Arabis alpina* formed another clade with *Arabidopsis* genus, suggesting independent evolutionary lineages occurred in Brassicaceae family (Fig 7A). In contrast, another phylogenetic tree was constructed without *Arabis alpina*, and pseudogene *rps16* of *A*. *stellari*, *A*. *hirsuta*, and *D*. *nemorosa* were observed to form one clade and remaining species containing intact *rps16* gene to form another clade (Fig 7B). However, Roy et al. [44] studied evolution of the *rps16* gene in the *Arabidopsis* and its closely related species, and commented phylogenetic tree construction with only one gene is unreliable and can misrepresent phylogenetic relationships, since a pseudogene does not always reflect the phylogenetic position of species. Therefore, it is possible gene or intron loss of *rps16* might have occurred independently in each species rather than by dependent evolution, which is supported by reports of independent *rps16* loss in *Medicago truncatula* [3], *Phaseolus vulgaris* [6], *Cicer arietinum* [47], *Vigna radiata* [48], and *Populus* genus [49, 50].

Additionally, we investigated the presence of *infA* protein-coding gene in Brassicaceae. The plastome gene, *infA* was completely absent in Brassicaceae family, which might have acquired a copy of the *infA* gene from either nuclear or mitochondrial genomes. Earlier studies also suggest that the gene *infA* have been lost in the Brassicales, Cucurbitales, Fabales, Fagales, Malphighlales, Malvales, Myrtales, Rosales, Sapindales, Solanales, *Dianthus* and *Lychinis* [6, 8–12].

### Evolution of the *ycf15* gene

**Fig 8:**
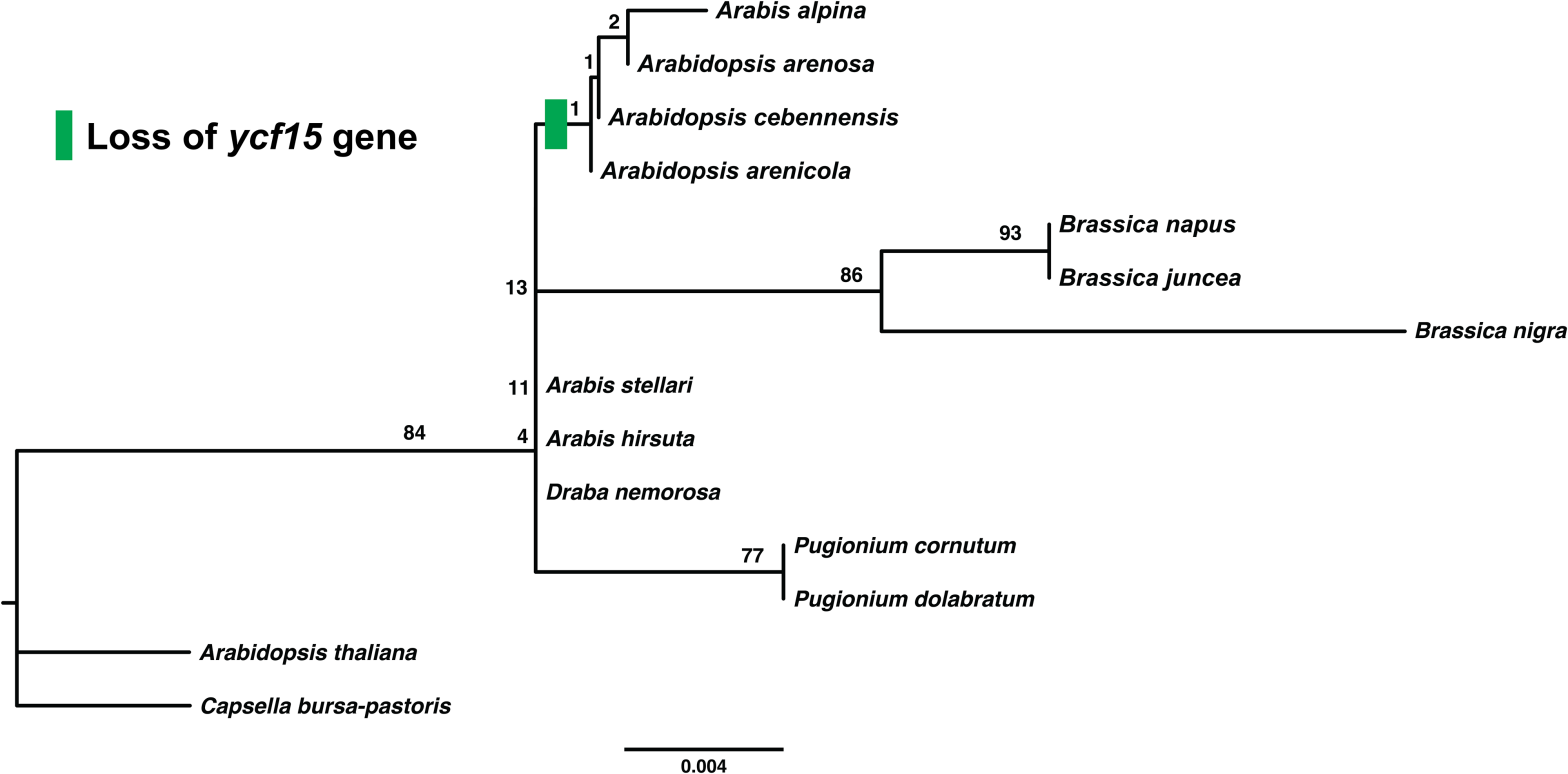
Molecular phylogenetic tree analysis of the cp protein-coding gene *ycf15* of Brassicaceae family. The tree was constructed by maximum likelihood (ML) analysis using the RaxML program and the GTRGAMMA nucleotide model. The stability of each tree node was tested by bootstrap analysis with 1000 replicates.

The plastome gene, *ycf15* encodes an ATG start codon in all species of Brassicaceae, suggesting it is probably a functional gene in this family. The genuses *Arabis*, *Draba*, *Capsella* and *Brassica* encode two intact copies of the 234-bp*yfc15* gene in their plastomes. *Pugionium* genus encoded only 162-bp for the *yfc15* gene, which may have been due to a point mutation (GAA to TAA) at the 160-bp position. Interestingly, in *Arabidopsis* genus, only *A*. *thaliana* encoded an intact *ycf15* gene, whereas other species, such as, *A*. *arenicola*, *A*. *arenosa* and *A*. *cebnnensis* encoded multiple internal stop codons, suggesting *ycf15* is disabled in these species (S3 Fig). However, comparative analysis suggested the organelle-encoded gene differs within the genus *Arabidopsis*. Nevertheless, the pseudogene, *ycf15* in these species might be transferred to the nucleus. Previous studies have also reported that internal stop codons in the *ycf15* gene of many angiosperms [51] and suggested that gene transfer from plastid to nucleus occurred more frequently during plastid evolution [52–54]. We also studied evolution of the *ycf15* gene in Brassicaceae (Fig 8). The evolutionary patterns of *ycf15* showed that it evolved independently in Brassicaceae species. Also, it contained an intact, an internal stop codon, or completely disabled or absent in the Brassicaceae phylogeny. Although, the same results were obtained when evolution of the *ycf15* gene was investigated in an angiosperm phylogenetic study [51].

### Synonymous (K_S_) and nonsynonymous (K_A_) substitution rate analysis

**Fig 9:**
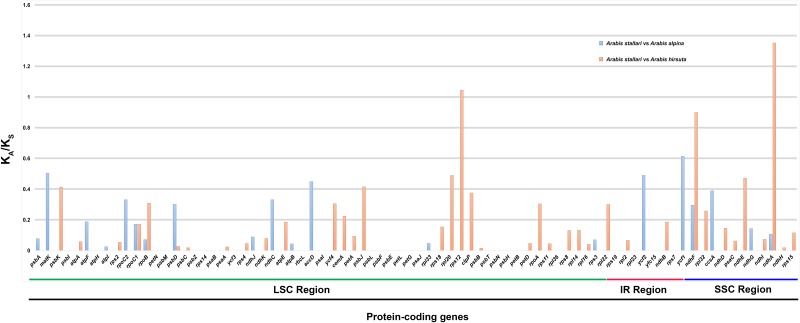
K_A_/K_S_ values of 79 protein-coding genes of *Arabis*. Blue color boxes indicate K_A_/K_S_ ratio of *A*. *stellari* vs. *A*. *alpina*, and orange boxes indicate those of *A*. *stellari* vs. *A*. *hirsuta*.

Synonymous and nonsynonymous nucleotide substitution patterns are more important indicators in gene evolution studies [55]. Although nonsynonymous substitutions occur much less frequently than synonymous substitutions, K_A_/K_S_ ratios are less than one in the majority of protein-coding genes [56]. In the present study, synonymous and nonsynonymous substitution rates were analyzed for 78 protein-coding genes of *A*. *stellari*, *A*. *alpina*, and *A*. *hirsuta* chloroplast genomes (Fig 9). The K_A_/K_S_ ratio of all genes was less than 1, except for *ndhA* of *A*. *hirsuta*. The K_A_/K_S_ ratio of *ndhA* of *A*. *stellari* vs. *A*. *hirsuta* was 1.35135. This deviation from unity was due to a four-amino acid change by nonsynonymous substitution and the deletion of five amino acids in the second exon of the *ndhA* gene of *A*. *stellari* due to silent mutation. Though, *ndhA* nucleotide identity was 98.2% vs. *A*. *hirsuta*. Although, the plastid genes, *atpH*, *petB*, *petG*, *petL*, *petN*, *psaB*, *psaI*, *psbE*, *psbF*, *psbH*, *psbI*, *psaJ*, *psbL*, *psbM*, *psbN*, *psbT*, *psbZ*, *rbcL*, *rpl23*, *rpl36*, *rps7*, *rps14*, *rps19*, *ycf3* and *ycf15* showed no synonymous or nonsynonymous changes occurred in the cp genomes of *A*. *stellari*, *A*. *alpina*, and *A*. *hirsuta*.

### Phylogenetic analysis of *A*. *stellari*

**Fig 10:**
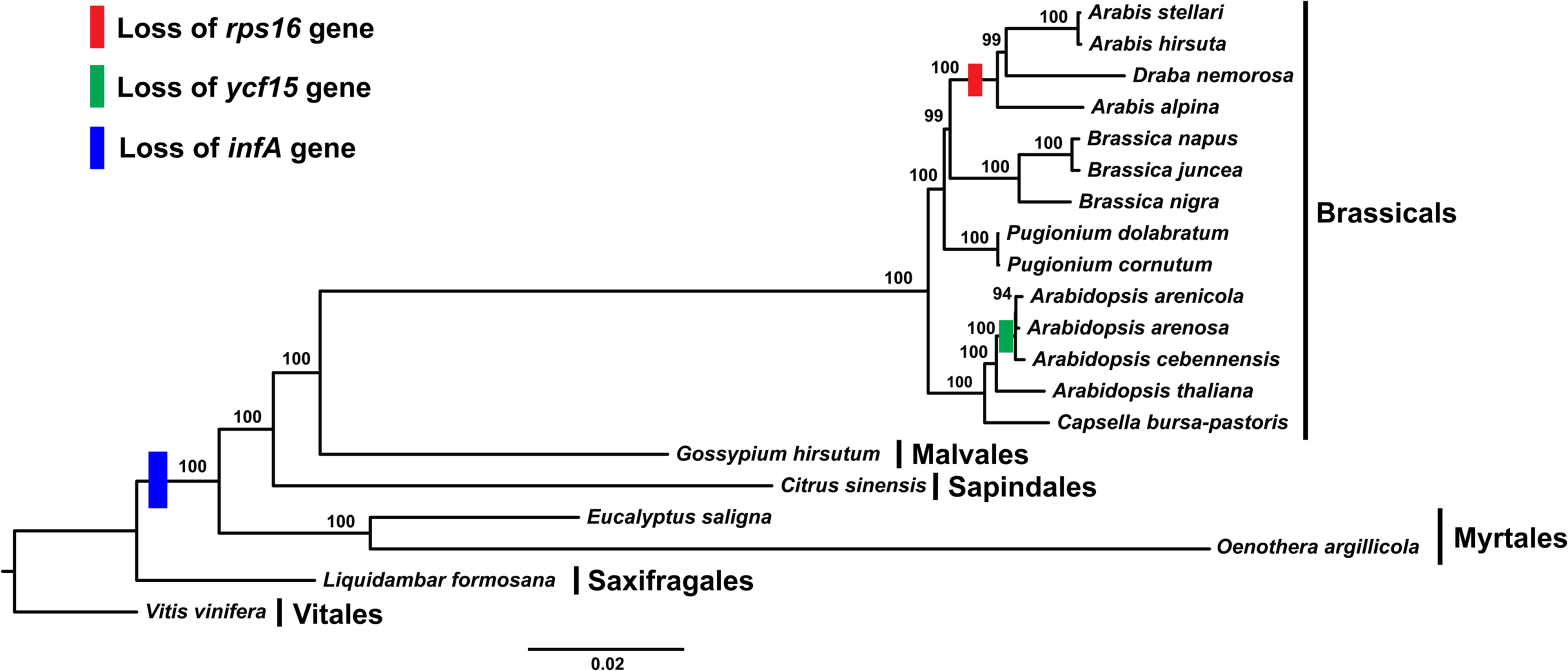
Molecular phylogenetic tree analysis of 76 cp protein-coding genes of Brassicaceae family. The tree was constructed by maximum likelihood (ML) analysis using the RaxML program and the GTRGAMMA nucleotide model. The stability of each tree node was tested by bootstrap analysis with 1000 replicates. *Vitis* was used as the outgroup.

To study the phylogenetic position of *A*. *stellari* within the Brassicaceae family, we used 76 protein-coding genes shared by the chloroplast genomes of 20 rosids and *Vitis* using the *Liquidambar* set as outgroups. Phylogenetic analysis revealed that Brassicaceae family formed a monophyletic group (Fig 10). *A*. *stellari* clustered with *A*. *hirsuta* with a bootstrap value of 100%, and *A*. *stellari* and *A*. *hirsuta* formed a sister clade with *D*. *nemorosa* rather than with *A*. *alpina*. Ten species of the Brassicaceae family showed extremely conserved chloroplast genome structures and their phylogenetic positions remained unaltered.

Overall, in the present study, we have compared the pseudogenization of *rps16*, *ycf15* and *infA* genes of Brassicaceae family. Fig 10 showed that the pseudogenization of *rps16* occurred only in *Arabis* genus whereas *ycf15* gene lost has not occurred in the entire genus of *Arabidopsis*. It occurred only in the species of *A*. *arenicola*, *A*. *arenosa* and *A*. *cebennensis*. While, the *infA* gene has lost in the entire Brassicals, Malvales, Sapindales and Myrtales. Based on these analysis, it suggested that the pseudogenization or gene lost event must have occurred in the species of *A*. *arenicola*, *A*. *arenosa* and *A*. *cebennensis* and Brassicals, Malvales, Sapindales and Myrtales after the earliest divergence lineage of the rosids.

## Conclusions

The chloroplast genome *Arabis stellari* was sequenced, analyzed, and compared with closely related species. Its total genome was found to be 153,683 bp long with a GC content of 36.4%. Overall gene contents were similar and gene arrangements was found to be highly conserved in the Brassicaceae family. Minor divergences were observed in the protein-coding genes *matK*, *ycf1*, *ccsA*, *accD*, and *rpl22* and a total of 991 SSRs were also detected in the *A*. *stellari* plastome genome. The K_A_/K_S_ nucleotide substitution ratio of *ndhA* gene of *A*. *stellari* vs. *A*. *hirsuta* was 1.35135. Furthermore, the genes *infA* and *rps16* were completed deleted but the *ycf15* gene was retained in the *Arabis* genus, and phylogenetic evolutionary studies revealed these genes evolved independently. In addition, phylogenetic analysis showed that the Brassicaceae species are extremely highly conserved based on their phylogenetic positions. It is hoped this study will be found useful by those involved in *Arabis* species conservation and molecular phylogenetic studies of Brassicaceae.

## Supporting information

S1 Fig PCR amplification of the *rps16* gene of *Arabis stellari* var. *japonica*. (TIF)

S2 Fig Comparisons of the *rps16* genes of Brassicaceae family. (TIF)

S3 Fig Comparisons of the *ycf15* genes of Brassicaceae family. (TIF)

S1 Table Accession numbers of the chloroplast genome sequences used in this study. (DOCX)

S2 Table Comparison of cp genome size, %GC content and total number plastid genes of Brasscicaceae family. (DOCX)
