# Supplementary figures and images for: Characterization of the complete chloroplast genome of Arabis stellari and comparisons with related species
Source: PLoS One. 2017 Aug 15;12(8):e0183197. doi: 10.1371/journal.pone.0183197 (PMC5557495; doi:10.1371/journal.pone.0183197)

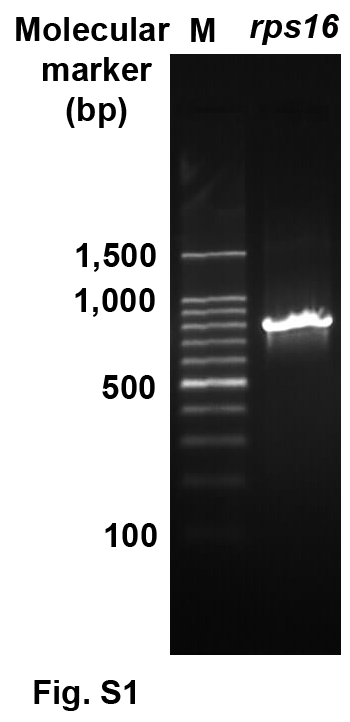

Supplement: S1 Fig — (TIF) [file pone.0183197.s001.tif]

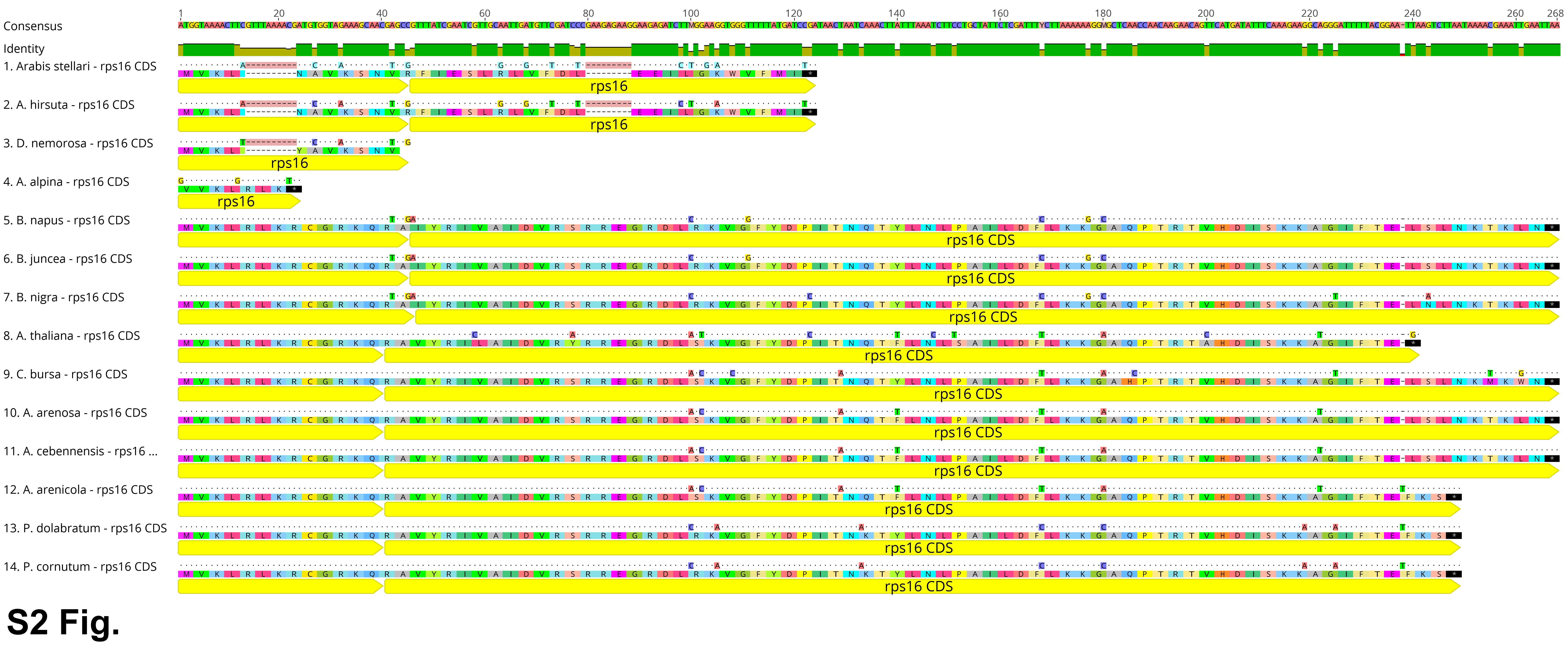

Supplement: S2 Fig — (TIF) [file pone.0183197.s002.tif]

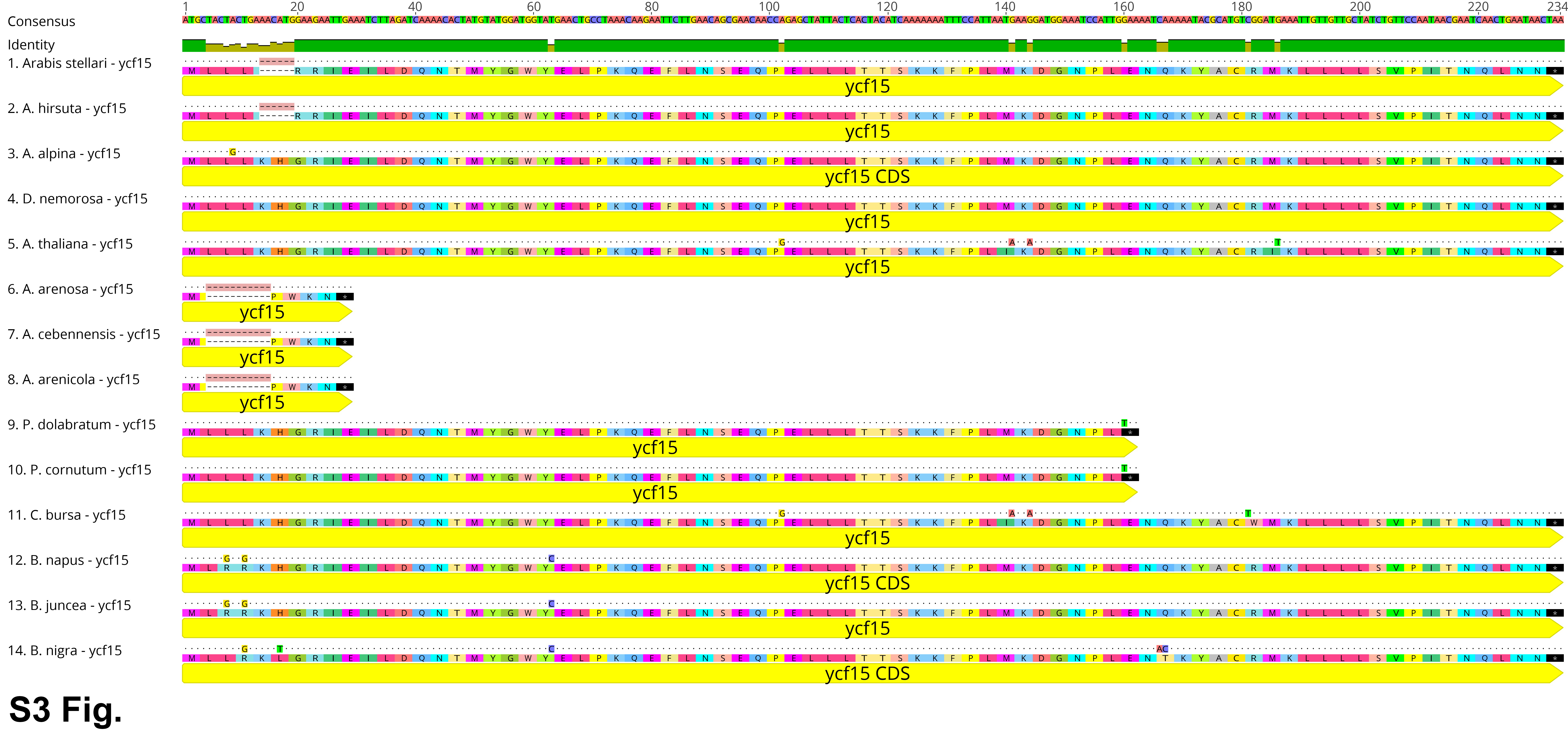

Supplement: S3 Fig — (TIF) [file pone.0183197.s003.tif]
