# Supplementary material for: Characterization of the complete chloroplast genome of Arabis stellari and comparisons with related species
Source: PLoS One. 2017 Aug 15;12(8):e0183197. doi: 10.1371/journal.pone.0183197 (PMC5557495; doi:10.1371/journal.pone.0183197)
Supplement: S1 Table — (DOCX) [file pone.0183197.s004.docx]

Table S1. List of the accession numbers of the chloroplast genome sequences used in this study.

| Sl. No. | Taxon | Family | Order | GenBank |
| --- | --- | --- | --- | --- |
| 10 | *Arabidopsis arenicola* | Brassicaceae | Brassicales | NC_030346 |
| 11 | *Arabidopsis arenosa* | Brassicaceae | Brassicales | NC_029334 |
| 12 | *Arabidopsis cebennensis* | Brassicaceae | Brassicales | NC_029335 |
| 13 | *Arabidopsis thaliana* | Brassicaceae | Brassicales | NC_000932 |
| 2 | *Arabis alpina* | Brassicaceae | Brassicales | NC_023367 |
| 3 | *Arabis hirsuta* | Brassicaceae | Brassicales | NC_009268 |
| 1 | *Arabis stellari* | Brassicaceae | Brassicales | KY126841 |
| 6 | *Brassica juncea* | Brassicaceae | Brassicales | NC_028272 |
| 5 | *Brassica napus* | Brassicaceae | Brassicales | NC_016734 |
| 7 | *Brassica nigra* | Brassicaceae | Brassicales | NC_030450 |
| 14 | *Capsella bursa-pastoris* | Brassicaceae | Brassicales | NC_009270 |
| 16 | *Citrus sinensis* | Rutaceae | Sapindales | NC_008334 |
| 4 | *Draba nemorosa* | Brassicaceae | Brassicales | NC_009272 |
| 17 | *Eucalyptus saligna* | Myrtaceae | Myrtales | NC_022397 |
| 15 | *Gossypium hirsutum* | Malvaceae | Malvales | NC_007944 |
| 19 | *Liquidambar formosana* | Altingiaceae | Saxifragales | NC_023092 |
| 18 | *Oenothera argillicola* | Onagraceae | Myrtales | NC_010358 |
| 9 | *Pugionium cornutum* | Brassicaceae | Brassicales | NC_030516 |
| 8 | *Pugionium dolabratum* | Brassicaceae | Brassicales | NC_030515 |
| 20 | *Vitis vinifera* | Viaceae | Vitales | NC_007957 |
