# Supplementary material for: Characterization of the complete chloroplast genome of Arabis stellari and comparisons with related species
Source: PLoS One. 2017 Aug 15;12(8):e0183197. doi: 10.1371/journal.pone.0183197 (PMC5557495; doi:10.1371/journal.pone.0183197)
Supplement: S2 Table — (DOCX) [file pone.0183197.s005.docx]

**Supplementary Table 2.** Comparison of cp genome size, %GC content and total number plastid genes of Brasscicaceae family.

| S. No. | Name of the species | Total cp genome size (bp) | GC content (%) | Total number of genes* | Total number of protein-coding genes | Total number of transfer RNAs | Total number of ribosomal RNAs |
| --- | --- | --- | --- | --- | --- | --- | --- |
|  | *Arabis stellari* | 153,683 | 36.4 | 113 | 79 | 30 | 4 |
|  | *Arabis hirsuta* | 153,689 | 36.4 | 113 | 79 | 30 | 4 |
|  | *Arabis alpina* | 152,866 | 36.4 | 113 | 79 | 30 | 4 |
|  | *Draba nemorosa* | 153,289 | 36.5 | 113 | 79 | 30 | 4 |
|  | *Brassica juncea* | 153,483 | 36.4 | 114 | 80 | 30 | 4 |
|  | *Brassica napus* | 152,860 | 36.3 | 114 | 80 | 30 | 4 |
|  | *Brassica nigra* | 153,633 | 36.4 | 114 | 80 | 30 | 4 |
|  | *Pugionium cornutum* | 154,992 | 36.2 | 114 | 80 | 30 | 4 |
|  | *Pugionium dolabratum* | 155,002 | 36.2 | 114 | 80 | 30 | 4 |
|  | *Arabidopsis arenicola* | 154,610 | 36.4 | 113 | 79 | 30 | 4 |
|  | *Arabidopsis arenosa* | 154,871 | 36.4 | 113 | 79 | 30 | 4 |
|  | *Arabidopsis cebennensis* | 154,504 | 36.4 | 113 | 79 | 30 | 4 |
|  | *Arabidopsis thaliana* | 154,478 | 36.3 | 114 | 80 | 30 | 4 |
|  | *Capsella bursa-pastoris* | 154,490 | 36.6 | 114 | 80 | 30 | 4 |

* Duplicated genes in the IR regions were not included.
